# P-cresol Alters Brain Dopamine Metabolism and Exacerbates Autism-Like Behaviors in the BTBR Mouse

**DOI:** 10.3390/brainsci10040233

**Published:** 2020-04-13

**Authors:** Tiziana Pascucci, Marco Colamartino, Elena Fiori, Roberto Sacco, Annalisa Coviello, Rossella Ventura, Stefano Puglisi-Allegra, Laura Turriziani, Antonio M. Persico

**Affiliations:** 1 Department of Psychology and Centro "Daniel Bovet", Sapienza University of Rome, I-00185 Rome, Italy; tiziana.pascucci@uniroma1.it (T.P.); marco.colamartino89@gmail.com (M.C.); elenafiori@hotmail.it (E.F.); lisax89@yahoo.it (A.C.); rossella.ventura@uniroma1.it (R.V.); 2 IRCCS Fondazione Santa Lucia, I-00143 Rome, Italy; 3 European Brain Research Institute EBRI, I-00161 Rome, Italy; 4 Service for Neurodevelopmental Disorders & Laboratory of Molecular Psychiatry and Neurogenetics, University “Campus Bio-Medico”, I-00128 Rome, Italy; r.sacco@unicampus.it; 5 IRCCS Neuromed, I-86077 Pozzilli, Italy; s.puglisiallegra@gmail.com; 6 Interdepartmental Program “Autism 0-90”, “Gaetano Martino” University Hospital, University of Messina, I-98125 Messina, Italy; turrizianilaura@gmail.com

**Keywords:** autism spectrum disorder (ASD), biomarker, *p*-cresol, mouse social behavior, dopamine

## Abstract

*Background:* Autism Spectrum Disorder (ASD) is a neurodevelopmental disorder characterized by deficits in social interaction/communication, stereotypic behaviors, restricted interests, and abnormal sensory-processing. Several studies have reported significantly elevated urinary and foecal levels of *p*-cresol in ASD children, an aromatic compound either of environmental origin or produced by specific gut bacterial strains. *Methods:* Since *p*-cresol is a known uremic toxin, able to negatively affect multiple brain functions, the present study was undertaken to assess the effects of a single acute injection of low- or high-dose (1 or 10 mg/kg i.v. respectively) of *p*-cresol in behavioral and neurochemical phenotypes of BTBR mice, a reliable animal model of human ASD. *Results:* P-cresol significantly increased anxiety-like behaviors and hyperactivity in the open field, in addition to producing stereotypic behaviors and loss of social preference in BTBR mice. Tissue levels of monoaminergic neurotransmitters and their metabolites unveiled significantly activated dopamine turnover in amygdala as well as in dorsal and ventral striatum after *p*-cresol administration; no effect was recorded in medial-prefrontal cortex and hippocampus. *Conclusion:* Our study supports a gene x environment interaction model, whereby *p*-cresol, acting upon a susceptible genetic background, can acutely induce autism-like behaviors and produce abnormal dopamine metabolism in the reward circuitry.

## 1. Background

Autism Spectrum Disorder (ASD) is a neuropsychiatric disorder that begins early in childhood and is characterized by deficits in social interaction and communication, repetitive behaviors, restricted interests, and abnormal sensory processing [1]. The incidence of ASD has dramatically risen during the last few decades, reaching the rate of 1 affected in 58 children [2], making autism one of the most widespread disorders in child neuropsychiatry [3,4]. Both genetic and environmental factors contribute to the pathogenesis of ASD [5,6]. A wide variety of environmental factors have been hypothesized to contribute to ASD pathogenesis, but conclusive evidence has been reached for a small minority, including prenatal infections, some medications (valproic acid, thalidomide, misoprostol, selective serotonin reuptake inhibitors), pesticides, and air pollutants, among others [7].

The complexity of ASD has spurred interest into patient subgrouping strategies, either based on endophenotyping or on biomarkers. Endophenotypes represent familial, heritable and quantitative traits associated with a complex disorder [8,9]. Biomarkers are associated with the disease without necessarily displaying heritability and familiarity; rather, they merely tag for the presence/absence of the disease due to environmental or pathophysiological links, not necessarily of a genetic nature [9]. A reliable set of autism biomarkers could foster earlier and more reliable diagnoses, predict developmental trajectories and treatment response, and identify individuals at high-risk, eventually leading to the establishment of preventive health care strategies, contributing to dissect ASD into more discrete clinical entities, and perhaps even revealing unknown causes of autism, at least in some cases [9].

In recent years, targeted and unbiased metabolomic studies have unveiled a set of potential ASD biomarkers, i.e., small urinary molecules significantly elevated in autistic children [10,11]. Among urinary solutes, *p*-cresol was found to be significantly elevated in autistic children compared to sex- and age-matched controls up until age 8, in two independent samples recruited in Italy and France [12,13]. This finding was later replicated measuring foecal *p*-cresol levels [14,15]. Using an unbiased approach, mass spectrometry-based urinary metabolomics detected *p*-cresol among the 20 solutes best able to differentiate small ASD children from matched controls [11]. Interestingly, elevated urinary *p*-cresol levels were significantly associated with chronic constipation in autistic children, pointing toward slow intestinal transit time as one the main factors allowing greater gut absorption of potentially neuroactive compounds, such as *p*-cresol [16]. The identification of *p*-cresol and of its metabolite *p*-cresylsulphate as two well-known neuroactive uremic toxins poses the question whether, aside from representing a potentially valuable biomarker, the consistent elevation of urinary *p*-cresol detected in young autistic children with chronic constipation may contribute to the clinical severity of their ASD [17]. Preliminary data point toward possible correlations between urinary *p*-cresol concentrations and ASD severity measured using the Childhood Autism Rating Scale (CARS) [12]. Multiple mechanisms could account for the negative influences of *p*-cresol on neural function, ranging from membrane depolarization and increased susceptibility to seizures [18], to decreased Na^+^-K^+^ ATPase activity [19], to blunted conversion of dopamine (DA) to norepinephrine (NE) due to inhibition of dopamine-β-hydroxylase [20].

The studies summarized above spur interest into testing *p*-cresol for behavioral effects in animals carrying a genetic predisposition toward autism-like behaviors. Despite several difficulties in developing rodent models with autistic features [21,22], to date, environmental, genetic, and lesion murine models reproducing autism-like behaviors have been developed [22,23,24,25,26]. The present study aims to assess the effects of acute *p*-cresol in a well-established inbred murine model of ASD, the BTBR mouse [23,27,28]. A single low dose of *p*-cresol (1 mg/kg) significantly raises anxiety and hyperactivity, two frequent ASD comorbidities, while acute administration of a higher dose (10 mg/kg i.v.) also exacerbates core symptoms of ASD, blunting interest in a conspecific intruder and enhancing stereotypic behaviors. Brain region-specific neurochemical analyses link these behaviors to parallel, dose-dependent increases in DA turnover in the AMY, nucleus accumbens (NAc) and dorsal caudate putamen (CP).

## 2. Methods

### 2.1. Animals

Every precaution was taken to minimize animal suffering and the number of animals used. For this study, only BTBR T+tf/J male mice were used. Parental strains were obtained from the Jackson Laboratories (Bar Harbor, ME, USA). After weaning at postnatal day (PND) 28, animals were housed 4 per standard breeding cage with food and water ad libitum on a 12:12 h dark:light cycle (lights on 07:00 a.m.–07:00 p.m.). Only male mice were included in the study to avoid possible variability, due to hormonal fluctuations in female mice. Behavioral experiments were carried at PND 60–70 and were performed on the second part of the day (h 01:00 p.m.–06:00 p.m.). Behavioral tests were performed blind to treatment. Mice were habituated to the behavioral testing room for 1 hour before starting the experiment. Tests were conducted in a sound-attenuated room and recorded through a camera (SSCDC378P, Sony, Tokyo, Japan) connected to a computer. Video were analyzed using the EthoVision video tracking software and the Observer XT program (Noldus information technology, Wageningen, The Netherlands) for automatic and manual recording, respectively.

All groups (CNTR, PC1 and PC10) were submitted to the elevated plus maze, open field motor test, object recognition test [29], and three-chamber social interaction test [30,31], in this order. Behavioral testing was performed 15 min after receiving a *p*-cresol/saline injection. Animals were sacrificed by rapid decapitation 100 min after the injection, heads were frozen and brains were removed and prepared for biochemical assay [32,33].

All experiments of this study were approved by the ethics committee of the Italian Ministry of Health and therefore conducted under license/approval ID #: 10/2011-B, according with Italian regulations on the use of animals for research (legislation DL 116/92) and NIH guidelines on animal care.

### 2.2. P-cresol Treatment

P-cresol was purchased from Sigma-Aldrich (St. Louis, MO, USA), dissolved in saline (0.9% NaCl) and the two different doses (1 or 10 mg/kg) were intravenously delivered by tail vein injection through a micro-cannula to reduce the stress of manipulation. Mice were randomly assigned to experimental groups: (a) naïve, (b) saline-treated controls, and (c) animals that received *p*-cresol 1 mg/kg (P-C1) or (d) *p*-cresol 10 mg/kg (P-C10). Since no difference was recorded between naïve and saline-treated animals, they were grouped together and defined as “control group” (CNTR). Behavior was tested 15 min after the injection.

### 2.3. Elevated Plus Maze

Emotional reactivity and anxiety-like behaviors were measured using the Elevated Plus Maze, a gray plexiglass apparatus with two open arms (27 × 5 cm) and two enclosed arms (27 × 5 × 15 cm) extending from a central platform (5 × 5 cm).

Animals were individually tested for 5 min, and the total number of entries in the open and closed arms, the percentage of entries in the open arms [(open entries/open + closed entries) × 100] and percentage of time spent in the open arms [(time in open arms/time in open + closed arms) × 100] were automatically analyzed using the EthoVision software.

### 2.4. Open Field Test 

The apparatus consists in a circular open field, 60 cm in diameter and 20 cm in height. Mice were individually introduced in the empty apparatus and left free to explore the arena for 30 min. Videos from each 30-min Open Field Test session were recorded. Distance travelled (cm) and speed (cm/s) were automatically analyzed using the EthoVision software. 

### 2.5. Object Recognition Test 

**Figure 1:**
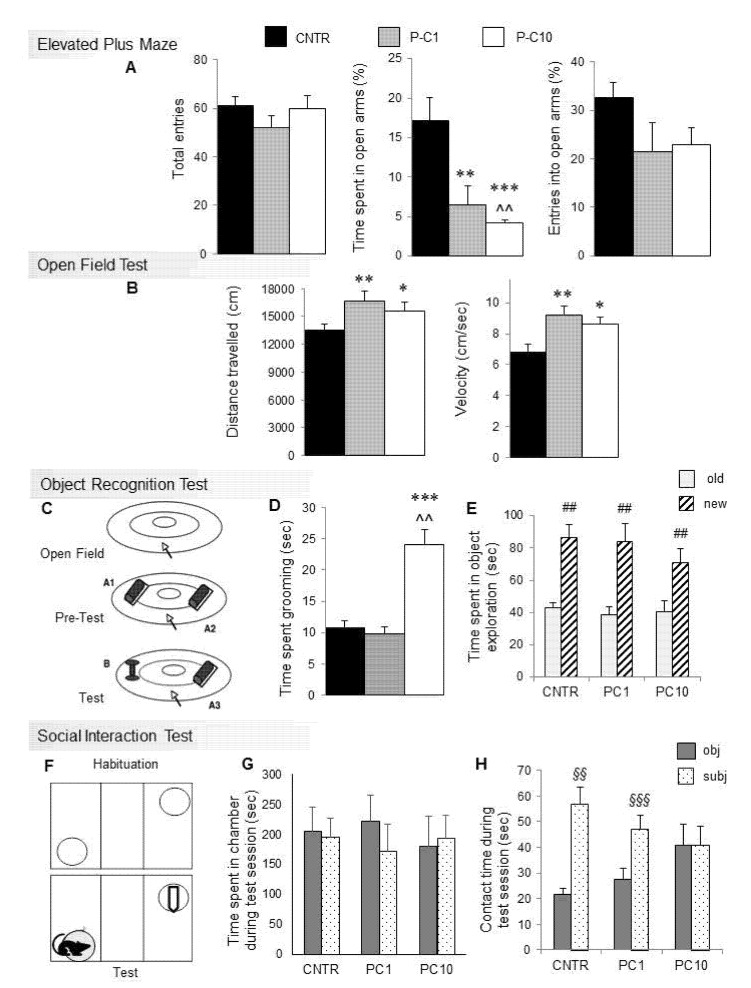
P-cresol enhances anxiety-like behaviors, stereotypies, locomotor parameters and hinders social preference in BTBR mice. (**A**) Total entries, % of time spent and entries in open arms in the Elevated Plus Maze. (**B**) Distance travelled and speed in the Open Field Test after acute *p*-cresol treatment. (**C**) Schematic representation of the Object Recognition Test. (**D**) Time spent grooming during the first session of the Object Recognition Test. (**E**) Time spent exploring the novel or familiar object during the test session of the Object Recognition Test. (**F**) Schematic representation of the three-chamber Social Interaction Test. (**G**) Time in object and subject zones during the Social Interaction Test session. (**H**) Time spent in contact with the object or with the social intruder during the Social Interaction Test. Results are shown as mean ± sem. *, **, *** *p* < 0.05, *p* < 0.01, *p* < 0.001 P-C1 or P-C10 vs. CNTR. ^^ *p* < 0.01 P-C10 vs. P-C1, ^##^ *p* < 0.01 old vs. new, ^§§^, ^§§§^ *p* < 0.01, *p* < 0.001 subject vs. object.

The apparatus is the same as for the Open Field Test (Figure 1C). Each mouse was individually submitted to three 6-minute sessions (Open Field, Pre-Test and Test sessions). At the end of each session, the animal was returned to its home cage for 3 min. All sessions were videotaped and analyzed by an experimenter trained to the Noldus Observer XT event coding software.

During the Open Field session, each mouse was left free to explore the arena for 6 min and time spent grooming was measured.

During the pre-Test session, the mouse was introduced in the arena containing two identical objects (A1 and A2: two identical black plastic cylinders of 8 cm in height and 4 cm in diameter, horizontally fixed to a rectangular base), as shown in Figure 1C, and left free to explore. Total time spent exploring two identical objects (A1 and A2) was measured and analyzed.

For the Test session, both objects were substituted, one with object A3, identical to the previous objects, and the other with the new object B (a red and gray plastic spool: 8 cm in height and 5 m in diameter). Object recognition was evaluated by comparing total time spent exploring the novel (B) vs. the familiar (A3) object. 

### 2.6. Three-chamber Social Interaction Test

The apparatus was a three-chamber box made in plexiglass (Figure 1F). Two transparent partitions (23 cm in height) with removable openings divided the box into three identical rectangular chambers (60 cm × 40 cm). The two external chambers contained two perforated plexiglass cylinders, used to enclose stranger BTBR mice. The test consisted in two 10 min sessions, encompassing the Habituation session and the Sociability Test session. Immediately after the Habituation session the animal was confined to the center chamber while an unfamiliar strain-, sex-, and age-matched adult intruder (subject) or an object were placed inside the cylinders. Videos were recorded and analyzed both automatically and manually, using the EthoVision and Observer XT programs. Time spent in each chamber, time spent in contact with the two cylinders, distance travelled and speed were recorded and analyzed.

### 2.7. Biochemical Assay

Biochemical assays were performed as previously described [32,33]. Briefly, frozen brains were fixed vertically on the freezing microtome pate. Punches were obtained from 300 μm-thick brain slices (coronal sections). Stainless steel tubes of 0.8, 1.0, or 1.5 mm inside diameter were used. Coordinates were measured as follows: medial pFC, two slices from section 80 to section 130 (1.5 mm tube); NAc, three slices from section 151 to section 201 (1.0 mm tube); CP, 4 slices from section 151 to section 230 (1.5 mm tube); AMY, 5 slices from section 251 to section 350 (0.8 and 1.0 mm tube); HIP, 3 slices from section 301 to section 350 (0.8 and 1.0 mm tube; including CA1, CA2 and CA3 fields). Punches were stored in liquid nitrogen until the day of analysis. Frozen tissues were then weighed and homogenized in 0.05 M HClO_4_. Homogenates were centrifuged at 14,000 rpm for 20 min at 4 °C. Tissue levels of DA, NE, 5-HT and their metabolites were assessed using HPLC. The HPLC system consists of an Alliance (Waters) system and a coulometric detector (ESA Model 5200A Coulochem II) provided with a 5011 high sensitivity analytical cell and a 5021 conditioning cell, the potential being set at 0.450 mV and 0.100 mV, respectively. A Nova-Pack Phenyl column and a Sentry Guard Nova-Pack pre-column were purchased from Waters Assoc. Flow rate was 1 ml/min. The mobile Phase consisted of 3% methanol in 0.1 M Na-phosphate buffer pH 3.0, 0.1 mM, Na_2_EDTA and 0.5 mM 1-octane sulphonic acid Na salt.

### 2.8. Statistical Analysis

Behavioral parameters recorded in the Elevated Plus Maze and Open Field Test were analyzed using one-way ANOVAs to detect group effects (three levels: CNTR, P-C1, P-C10), followed by a post-hoc Duncan’s test. For the Object Recognition Test, the total time spent exploring the familiar (A3) vs. the novel (B) object during the test session were analyzed by two-way ANOVA for repeated measures (“group”, three levels: CNTR, P-C1, P-C10 as between factor; “object”, two levels: A3 and B as within factor). Simple effect analysis of the factor “object” was also performed within each group. Similarly, for the Social Interaction Test time spent in each chamber and time spent in contact with the two cylinders were analyzed by two-way ANOVA for repeated measures (“group” three levels: CNTR, P-C1, P-C10 as between factor; “zone”, two levels: object and subject as within factor). Distance travelled and speed by treatment group were analyzed using one-way ANOVA, followed by Duncan’s post-hoc test. Data are presented as mean ± sem. 

One-way ANOVAs, followed by a post-hoc Duncan’s test, were used for statistical analysis of the effects of treatment (three levels: CNTR, P-C1, P-C10) for each amine and metabolite (ng/g wet weight) within each brain region.

## 3. Results

### 3.1. P-cresol Enhances Anxiety-like Behaviors in BTBR Mice

The Elevated Plus Maze test is based on the natural inclination of mice to avoid open, elevated and bright places, in spite of their tendency to actively explore novel environments. Results are shown in Figure 1A (CNTR, *n* = 10; P-C1, *n* = 8; P-C10, *n* = 8 mice). The percentage of time spent in the open arms by the CNTR group (17.13%) is consistent with previous studies [34]. P-cresol (1 and 10 mg/kg) profoundly decreases the percentage of time spent in the open arms (F_2,23_ = 10.632; *p* < 0.001), without significantly affecting the total number of entries (F_2,23_ = 1.187; *p* = 0.32) and the percentage of entries in the open arm (F_2,23_ = 1.644; *p* = 0.21). Hence, both low and high *p*-cresol doses increase anxiety-like behaviors in BTBR mice tested using the Elevated Plus Maze.

### 3.2. Locomotor Activity is Enhanced by p-cresol in the Open Field Test

Results from the Open Field Test are displayed in Figure 1B (CNTR, *n* = 10; P-C1, *n* = 9; P-C10, *n* = 7). Both low- and high-dose *p*-cresol significantly enhanced distance travelled (F_2,23_ = 5.826; *p* < 0.01) and speed (F_2,23_ = 5.914; *p* < 0.01) compared to control mice, already yielding hyperactivity at low *p*-cresol doses.

### 3.3. P-cresol Enhances Motor Stereotypies without Modifying Object Recognition and Discrimination Behaviors

During the first Object Recognition Test session (Figure 1C), time spent grooming was measured (CNTR, *n* = 8; P-C1, *n* = 7; P-C10, *n* = 7). Figure 1D shows that the P-C10 group spent significantly more time self-grooming compared with controls and P-C1 animals (F_2,19_ = 18.12; *p* < 0.001), who do not differ from each other. A partial dose-dependent shift from hyperactivity to stereotyped behaviors was thus recorded.

Time spent exploring two identical objects during the Pretest session of the Object Recognition Test did not differ between controls and treatment groups (mean ± sem: CNTR = 80.27 ± 6.59; PC-1 = 88.09 ± 6.25; PC-10 = 67.55 ± 11.92; F_2,23_ = 1.426 *p* = 0.264, data not shown), demonstrating unchanged interest in object exploration. Similar results were obtained during the Test session (Figure 1E), indicating that *p*-cresol does not significantly influence the ability to discriminate novel vs. familiar objects (F_2,19_ = 0.897; *p* = 0.424).

### 3.4. High Dose p-cresol Thwarts Preference for Social Interaction

Behavioral results from the three-chamber Social Interaction Test are displayed in Figure 1G,H (CNTR, *n* = 6; P-C1, *n* = 7; P-C10, *n* = 7). No treatment effect was recorded on general motor activity neither during the habituation session (distance travelled: F_2,16_ = 3.342; *p* = 0.054; speed: F_2,16_ = 1.544; *p* = 0.237; time spent in each chamber: F_2,16_ = 0.276; *p* = 0.763), nor during the Sociability Test session (distance travelled, F_2,16_ = 1.504; *p* = 0.243; speed: F_2,16_ = 1.572; *p* = 0.229; time spent in each chamber F_2,16_ = 0.164; *p* = 0.85) (Figure 1G). Time spent sniffing the cylinders did not differ during habituation (F_2,16_ = 0.263; *p* = 0.77), whereas a significant treatment effect was recorded during the Sociability Test over time spent in contact with the cylinders containing subject vs. object (F_2,16_ = 6.241; *p* < 0.01). In fact, CNTR and low-dose cresol-treated animals (P-C1) maintained a significant preference for the social stimulus, while high-dose cresol-treated animals (P-C10) lost their social preference, spending the same amount of time sniffing the two cylinders containing either the conspecific intruder or the object (Figure 1H).

### 3.5. P-cresol Enhances Dopamine Metabolism in NAc, CP and AMY

**Figure 2:**
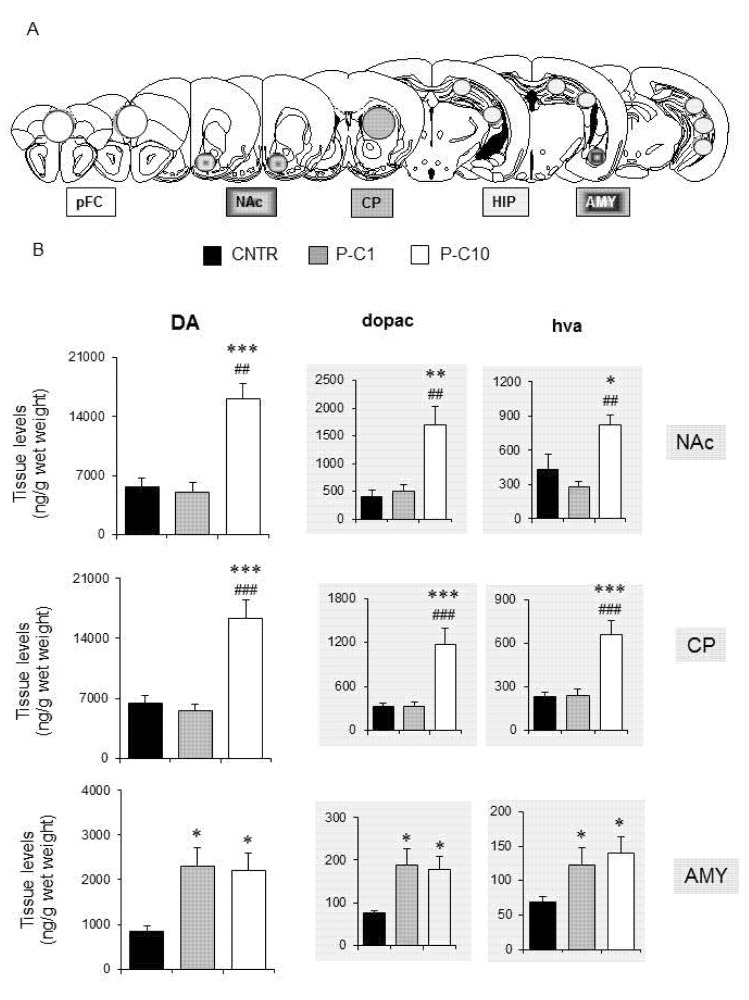
P-cresol enhances tissue levels of dopamine and its metabolites in the amygdala, caudate putamen and nucleus accumbens of BTBR mice. (**A**) Tissue levels of DA, DOPAC, HVA, NE, 5-HT and 5-HIAA, measured in medial pFC, NAc, CP, HIP, AMY. (**B**) Tissue levels of DA, DOPAC, HVA, measured in NAc, CP and AMY. CNTR, *n* = 9–10; P-C1, *n* = 6, P-C10 *n* = 6. Data are expressed as mean ± sem ng/g wet weight. *, **, *** *p* < 0.05, 0.01, 0.001 P-C1 or P-C10 vs. CNTR group. ^##^, ^###^ *p* < 0.01, 0.001 P-C10 vs. P-C1 (treatment effect) by Duncan’s post-hoc test following one-way ANOVAs. Abbreviations: AMY: Amygdala; CP: Caudate Putamen; DA: dopamine; DOPAC: 3,4-Dihydroxyphenylacetic acid; HIP: Hippocampus; HVA: Homovanillic acid; NAc: Nucleus Accumbens; pFC: preFrontal Cortex.

Neurochemical data concerning brain levels of monoamines and their metabolites assessed in medial pFC, HIPP, AMY, CP and NAc are summarized in Table 1 and Figure 2 (CNTR, *n* = 9; P-C1, *n* = 6; P-C10, *n* = 6). Significant treatment effects were recorded in NAc, CP and AMY on levels of DA (NAc F_3,18_ = 21.358; *p* < 0.001; CP: F_3,15_ = 13.028; *p* < 0.001; AMY: F_3,15_ = 3.267; *p* < 0.05), HVA (CP: F_3,15_ = 8.988; *p* < 0.001; NAc: F_3,18_ = 6.649; *p* < 0.01), and DOPAC (NAc: F_3,18_ = 9.886; *p* < 0.001; CP: F_3,15_ = 5.851; *p* < 0.001; AMY: F_3,15_ = 3.482; *p* < 0.05) (Figure 2B). DA turnover was largely enhanced in NAc and CP and only by high-dose *p*-cresol (P-C10); whereas in AMY, both low- and high-dose *p*-cresol were equally effective (Figure 2B). No significant change was recorded for norepinephrine and 5-HIAA, whereas 5-HT levels were increased only in the CP following the higher dose of *p*-cresol (F_2,16_ = 8.927; *p* < 0.01) (Table 1). No treatment effect was detected in medial pFC and HIPP for any monoamine or metabolite level (Table 1).

## 4. Discussion

In the present study, acute *p*-cresol administration to BTBR mice, a reliable animal model of ASD [23,27,28], elicited autism-like behaviors and enhanced dopaminergic turnover both in the AMY, and in the dorsal and ventral striatum. Importantly, behavioral abnormalities elicited by *p*-cresol in BTBR mice strikingly resemble core symptoms and co-morbid disorders clinically observed in human autistic individuals. On the one hand, excessive interest in objects over social interaction and stereotypic behaviors represent two of the hallmarks of an ASD diagnosis in humans [1]. Additionally, hyperactivity and anxiety are among the most frequent co-morbidities in autistic patients, with ADHD and anxiety disorders being diagnosed in 33%–37% and in 39.6% of ASD cases, respectively [35,36]. BTBR mice are an inbred strain spontaneously displaying autism-like behaviors [23,27,28]. These behavioral abnormalities likely stem from strain-specific genetic underpinnings involving neurodevelopmental genes, like kynurenine 3-hydroxylase (*Kmo*), Disrupted in Schizophrenia (*Disc1*) and exostosin 1 (*Ext1*) [28]. The induction of hyperactivity in the Open Field Test, but not in the 3-chamber Social Interaction Test, most likely represents only an apparent contradiction, because the more interesting social interaction apparatus is able to engage motivated exploratory behaviors in mice that can “cover” the spontaneous hyperactivity visible in the Open Field Test. In addition, differences in session duration between the two tests (30 min in the Open Field Test vs. 10 minutes in the Social Interaction Test) can further influence the expression of hyperactivity in treated BTBR. Instead, a large body of literature reports a lack of sociability in BTBR using the three-chambered social approach, although data showing that BTBR control mice display significant sociability [37,38,39,40] or a non-significant preference for subject exploration are also present (see Figure 1B in ref. [40], Figure 3B in ref. [41], and Figure 3B in ref. [42]). One possible explanation for these discrepancies is that genetically-driven ASD-like behaviors in the BTBR strain may spontaneously be under threshold and may emerge to a different extent depending upon experimental manipulations, handling or treatments [37]. Furthermore, discrepancies due to different choice of intruder (conspecific vs. different strain) in the Social Interaction Test cannot be excluded (in present study we used a BTBR conspecific intruder). Baseline control behavioral parameters recorded in our BTBR mice in the Elevated Plus Maze, Object Recognition Test and Social Interaction Test are absolutely in line with previous studies from our lab [29,32,43,44] and are coherent with the overall literature [45,46,47], although absolute values predictably differ, likely due to differences in housing environment, animal handling, and test settings. Finally, blunted social preference in the three-chamber test could conceivably stem from enhanced anxiety rather than reflecting a real social interaction deficit. While we cannot exclude contributions by anxiety to this behavior, the emotional reaction of BTBR mice to the objects during pre-test and test sessions of the Object Recognition Test did not differ between groups, as all groups spent the same time exploring objects. Most importantly, both low- and high-dose *p*-cresol produced anxiety-like behaviors in the Elevated Plus Maze. Therefore, if anxiety played a pivotal role in reducing social preference, the lower *p*-cresol dose should have also been effective. In summary, our results collectively support a gene x environment interaction model, whereby, acting upon a susceptible genetic background, *p*-cresol triggers anxiety and hyperactivity at a low dose, while boosting core autism-like symptoms at the higher dose.

Behavioral abnormalities are paralleled by neurochemical alterations, mainly involving the dopaminergic turnover. This interpretation is in line with long-standing evidence of dopamine-β-hydroxylase inhibition by *p*-cresol [20] and with the proportionate increase in DA and its metabolites, supporting increased DA accumulation, release and catabolism (both intra- and extra-cellular). However, the measurable, albeit non-significant, increase in NE recorded in several brain regions displaying increased DA and its metabolites (Table 1) indicates that enhanced DA synthesis may also contribute to cresol-induced dopaminergic imbalance. On the one hand, levels of DA and its metabolites were dose-dependently increased in the ventral and dorsal striatum, where only the higher *p*-cresol dose was effective (Figure 2B). On the other hand, dose-independent effects were recorded in the AMY, where low- and high-dose *p*-cresol were equally effective in boosting DA turnover (Figure 2B). This regional distribution and dose-dependency fit well with the pattern of behavioral abnormalities recorded in these same animals. Low- and high-dose *p*-cresol were equally effective in reducing time spent in the open arms at the Elevated-Plus Maze and in enhancing locomotor activity (Figure 1A,B). Instead, only high-dose *p*-cresol significantly increased stereotypic behaviors and blunted social interaction(Figure 1D,H). This trend resembles the effects of acute amphetamine in rodents, yielding hyperactivity at low doses and stereotypic behaviors (sniffing and grooming) at higher doses [48,49]. Drosophila melanogaster carrying the ASD-associated hDAT ΔN336 variant, which impairs DA uptake while sparing DA efflux, displays behavioral abnormalities that are strikingly overlapping with those recorded here following acute *p*-cresol—namely increased fear, impaired social interactions, and enhanced locomotion [50]. Modest increases in 5-HT levels parallel the much larger changes observed in levels of dopamine and its metabolites (Table 1). We cannot exclude synergistic serotoninergic contributions to cresol-induced behavioral effects, since 5-HT transporter KO mice display at least some autism-like behaviors, including social deficits and increased anxiety [51]. However, changes in brain 5-HT levels are relatively minor compared to changes in DA and never reach statistical significance, except in the striatum following high-dose *p*-cresol (Table 1). Furthermore, changes in 5-HIAA levels are even more modest, and there is only partial overlap between serotoninergic neurochemical parameters and behavioral changes. Collectively, serotoninergic contributions to cresol-induced behavioral abnormalities may seemingly play a secondary role at best. Instead, our data strongly reinforce the “dopamine hypothesis” of ASD [52], pointing toward the existence in autistic brains of two distinct dopaminergic activation thresholds: a lower threshold in the AMY to boost anxiety and hyperactivity, and a higher threshold in ventral and dorsal striatum to produce stereotypic behaviors and to divert motivational drives from interaction with conspecific animals to inanimate objects. D1 receptor activation or D2 receptor knock-out in the dorsal striatum have been shown to yield autistic-like behaviors in mice [53]. In line with this evidence, BTBR mice display blunted DRD2 signaling and responsiveness to extracellular DA in the presence of preserved DRD2 mRNA and protein levels [54]. On the other hand, comparable DRD1 expression and responsiveness to DA was recorded in BTBR and in C57Bl6 mice [54]. Altogether, much of the current literature on the motivational circuitry in ASD underscores reward-processing deficits towards social and monetary incentives [55,56]. Instead, results displayed in Figure 1H promote a more balanced view, whereby reduced DA activation by social stimuli may be seemingly paired with preserved or even enhanced DA activation by exposure to inanimate objects or by sensory self-stimulation [57,58,59]. Future experiments will have to extend the present findings, identifying the receptor and signaling pathways mediating the dopaminergic effects recorded in our experiments, and to explore whether the activation of DA turnover by *p*-cresol contributes to favoring LTP-based synaptic plasticity in the NAc [60], possibly fostering “addictive” attitudes towards routines, objects, or absorbing interests including internet and videogames.

Urinary and foecal levels of *p*-cresol have been consistently found elevated in autistic children compared to typically developing controls [11,12,13,14,15,16]. Preliminary evidence suggests that high urinary *p*-cresol may be clinically associated with greater autism severity and history of behavioral regression [12,17]. P-cresol is not produced by human cells, which lack *p*-hydroxyphenylacetate decarboxylase (pHPAD), the final enzyme of tyrosine transformation into *p*-cresol [17]. Hence, urinary *p*-cresol is either absorbed through the skin, the gut and the lungs from a variety of environmental sources (listed in Table 2 in ref. [17]), or it is produced by gut bacterial strains able to express pHPAD. The primary origin of urinary *p*-cresol elevation in autistic children remains to be determined, as does the reason for its normalization after age 8. However, its association with chronic constipation and longer intestinal transit time supports greater *p*-cresol absorption through the gut, while no association with the “leaky gut” was observed [16]. Chronic constipation thus likely represents a broad, non-specific facilitator of neurotoxic effects exerted by environmental and gut-derived compounds.

The present results raise further interest into *p*-cresol, not only as an ASD biomarker but also as a potential contributor to autism pathogenesis, by boosting DA turnover in specific brain regions of autistic individuals. P-cresol is certainly not the only neuroactive exogenous compound produced by gut bacteria and able to negatively affect behavior. Propionic acid, a short chain fatty acid produced by anaerobic gut bacteria including Clostridia and Propionibacteria, has been shown to produce a variety of behavioral, immune, mitochondrial effects in rodent models closely resembling human ASD [61]. Studies of urinary and foecal levels of propionic acid in autistic children compared to typically developing controls have yielded conflicting results [14,15]. Nonetheless, this compound could indeed play a pathoplastic role in specific patient subgroups, which need to be better defined at the clinical level. Meanwhile, additional tryptophan-derived gut bacterial compounds were found significantly elevated in the urines of autistic children, namely indolyl 3-acetic acid, indoxyl sulfate, and indolyl lactate [11]. These compounds have not yet been thoroughly assessed for possible neuroactive behavioral effects.

## 5. Limitations

The main limitation of the present study is the lack of a reversal experiment, showing that abnormal behaviors are corrected by administering dopamine receptor antagonists. Due to practical constraints, sample sizes of BTBR mice are relatively small, but 4–5 different litters were used for behavioral experiments and behavioral data appear reasonably consistent among different litters. In fact, all significant differences between control vs. cresol-treated animal mean values displayed in Figure 1 are at least three times larger than inter-litter S.E.M.s per each sample, with the sole exception of the Social Interaction Test (object vs. subject contact time) were P-C10 and controls differ 2.47 times the interlitter S.E.M. values of controls. Repetitive behaviors/restricted interests were assessed only by measuring stereotypic motor activity in the open field test, and not by applying specific tests designed to quantify mouse behaviors corresponding more closely to this diagnostic criterion. Locomotor activity data could have provided additional information if broken down into bins of 3–5 min, allowing an assessment of how quickly the mice habituate to the open field, and the time course of *p*-cresol effects. Finally, urinary baseline levels of endogenous *p*-cresol should be measured and compared among different inbred mouse strains because, if particularly elevated in BTBR mice, they could promote their autism-like phenotypic features and contribute to the behavioral abnormalities induced by exogenous *p*-cresol administration. In addition to addressing these limitations, our follow-up study will involve in parallel both the hypersociable C57Bl/6 mice and the ASD model BTBR mice, to further test the hypothesis that the behavioral abnormalities exacerbated by acute *p*-cresol are the result of a BTBR-specific gene x environment interaction. 

## 6. Conclusions

This study demonstrates that acute *p*-cresol administration to an animal model of ASD induces behavioral abnormalities closely resembling core symptoms of ASD and comorbidities frequently observed in autistic individuals. These results underscore the importance of gene x environment interaction models, able to merge genetic predisposition and evidence-based environmental exposure to specific neurotoxic compounds into a unitary scenario. From a mechanistic standpoint, these results move the field beyond well-established paradigms in the autism literature, such as the imbalance between glutamate and GABA to explain insistence on sameness and the co-morbidity with epilepsy [62], or the role of 5-HT in reference to hyperserotonemia, disruption of circadian rhythmicity, neuroinflammation and neuronal excitability [63,64,65]. In a complementary view, they point toward critical dopaminergic roles in autistic symptoms as being relevant as stereotypic behaviors, hyperactivity, anxiety and motivational drive towards inanimate objects. Thirdly, urinary gut-derived neurotoxic compounds, such as *p*-cresol, could serve as useful ASD biomarkers, whose specificity now deserves to be assessed in samples of young non-autistic children affected with chronic constipation. Finally, the correction of chronic constipation and microbiota transfer therapy represent two reasonable and testable approaches, aimed at partly ameliorating autistic behaviors by reducing the absorption of neurotoxic compounds of environmental origin or derived from specific gut-bacterial strains [66]. Studies addressing the efficacy of these therapeutic approaches will largely benefit from parallel assessments of urinary biomarkers, such as *p*-cresol and other gut-derived compounds, in order to provide mechanistic insights into their effects on the longitudinal time course of autistic symptoms.

**Availability of data and materials:** The datasets used and/or analyzed during the current study are available from the corresponding authors on reasonable request.

## Figures and Tables

**Table 1:** Neurochemical analysis of monoamine and metabolite levels (ng/g wet weight) assessed in medial prefrontal cortex, hippocampus, amygdala, caudate putamen and nucleus accumbens.

		DA	DOPAC	HVA	NE	5HT	HIAA
**pFC**	**CNTR**	342.95 ± 99.78	44.96 ± 9.21	43.95 ± 7.91	179.12 ± 26.24	826.28 ± 116.29	266.67 ± 51.36
	**P-C1**	305.53 ± 57.11	43.79 ± 11.19	37.07 ± 6.91	132.30 ± 25.35	607.64 ± 74.63	152.62 ± 15.13
	**P-C10**	423.87 ± 138.8	76.9 ± 24.84	42.91 ± 14.9	139.42 ± 36.39	881.03 ± 207.31	171.06 ± 39.93
**HIPP**	**CNTR**	155.18 ± 15.24	41.73 ± 15.71	29.98 ± 2.79	n.d.	641.22 ± 173.86	378.98 ± 59.82
	**P-C1**	113.92 ± 26.27	21.54 ± 7.92	22.77 ± 3.72	n.d.	425.17 ± 92.36	327.61 ± 102.74
	**P-C10**	119.47 ± 40.91	37.29 ± 12.97	30.65 ± 7.65	n.d.	365.6 ± 145.62	314.2 ± 99.42
**AMY**	**CNTR**	858.36 ± 112.78	68.98 ± 6.62	57.41 ± 11.05	274.34 ± 103.69	193.46 ± 42.02	149.84 ± 55.3
	**P-C1**	2292.71 ± 526.75 *	187.95 ± 38.11 *	122.05 ± 26.41 *	314.18 ± 89.95	356.62 ± 109.27	212.38 ± 88.17
	**P-C10**	2197.45 ± 992.31 *	179.38 ± 30.12 *	140.21 ± 23.75 *	283.15 ± 48.49	330.85 ± 59.27	226.03 ± 36.56
**CP**	**CNTR**	5284.18 ± 1015.8	584.97 ± 186.65	251.81 ± 33.64	35.46 ± 8.61	159.61 ± 31.77	133.99 ± 28.56
	**P-C1**	5499.14 ± 842.38	327.32 ± 56.27	240.48 ± 43.35	46.41 ± 8.61	183.88 ± 31.09	130.22 ± 38.39
	**P-C10**	16270.59 ± 2153.37 ***,^###^	1176.26 ± 223.34 ***,^###^	658.32 ± 97.93 ***,^###^	63.11 ± 12.83	341.1 ± 34.82 **,^##^	242.73 ± 49.04
**NAc**	**CNTR**	5623.89 ± 1050.64	506.55 ± 91.15	412.55 ± 87.32	1214.04 ± 390.84	1191.69 ± 355.05	459.72 ± 126.19
	**P-C1**	5035.05 ± 1134.2	504.3 ± 119.73	276.01 ± 49.36	1359.63 ± 200.81	1232.82 ± 357.02	305.25 ± 63.12
	**P-C10**	16156.11 ± 1812.97 **,^##^	1698.82 ± 325.04 **,^##^	817.35 ± 91.08 *,^##^	1883.69 ± 626.27	1360.47 ± 512.3	480.62 ± 189.73

Data are shown as mean ± sem. Highlighted in bold, significant effects of group x amine or metabolite. CNTR, *n* = 9; P-C1, *n* = 6; P-C10, *n* = 6. *, **, ***p < 0.05, 0.01, 0.001 P-C1 or P-C10 vs. CNTR. #, ##, ### *p* < 0.05, 0.01, 0.001 P-C10 vs. P-C1.

## Abbreviations

AMY	Amygdala
ASD	Autism Spectrum Disorder;
CARS	Childhood Autism Rating Scale;
CNT	Control;
CP	Caudate Putamen;
DA	dopamine;
DOPAC	3,4-Dihydroxyphenylacetic acid;
HIP	Hippocampus;
HVA	Homovanillic acid;
pFC	preFrontal Cortex;
NAc	Nucleus Accumbens;
NE	Norepinephrine;
5-HIAA	5-hydroxyindoleacetic acid;
5-HT	Serotonin,
WT	Wild-type
